# Combined Locked Nucleic Acid Probes and High-Resolution Melting Curve Analysis for Detection of Rifampicin-Resistant Tuberculosis in Northern Thailand

**DOI:** 10.3390/diagnostics12102307

**Published:** 2022-09-24

**Authors:** Yee Mon Thant, Sukanya Saikaew, Chayada Sitthidet Tharinjaroen, Ponrut Phunpae, Rodjana Pongsararuk, Kanya Preechasuth, Bordin Butr-Indr, Sorasak Intorasoot, Khajornsak Tragoolpua, Angkana Chaiprasert, Usanee Wattananandkul

**Affiliations:** 1 Division of Clinical Microbiology, Department of Medical Technology, Faculty of Associated Medical Sciences, Chiang Mai University, Chiang Mai 50200, Thailand; 2 Faculty of Public Health, Chiang Mai University, Muang District, Chiang Mai 50200, Thailand; 3 Infectious Diseases Research Unit (IDRU), Faculty of Associated Medical Sciences, Chiang Mai University, Muang District, Chiang Mai 50200, Thailand; 4 Office of Disease Prevention and Control Region, 1 Chiang Mai, Department of Disease Control, the Ministry of Public Health, Muang District, Chiang Mai 50000, Thailand; 5 Office for Research and Development, Faculty of Medicine Siriraj Hospital, Mahidol University, Bangkok 10700, Thailand; 6 Epidemiology Research Group of Infectious Disease (ERGID), Faculty of Nursing, Chiang Mai University, Chiang Mai 50200, Thailand

**Keywords:** tuberculosis, diagnosis, molecular test, real time PCR, single nucleotide polymorphism

## Abstract

Rifampicin-resistant tuberculosis (RR-TB) has become a major threat globally. This study aims to develop a new assay, RIF-RDp, to enhance the detection of RR-TB based on combined locked nucleic acid (LNA) probes with high-resolution melting curve analysis (HRM). Two new LNA probes were designed to target the class-III and IV mutations of *rpoB*, H526D, and D516V. LNA probes showed 100% specificity in the detection of mutant targets among characterized and blinded *Mycobacterium tuberculosis* (Mtb) isolates. The performance of RIF-RDp was evaluated using 110 blinded clinical Mtb isolates in northern Thailand against drug-susceptibility testing (DST), DNA sequencing, and a commercial real-time PCR kit. This assay showed sensitivity and specificity of 94.55% and 98.18% compared to DST, and 96.36% and 100% compared to DNA sequencing. The efficacy of RIF-RDp was comparable to the commercial kit and DNA sequencing. The Cohen’s Kappa statistic showed almost perfect agreement between RIF-RDp and the commercial kit (κ = 0.95), and RIF-RDp and DNA sequencing (κ = 0.96). Furthermore, this is the first report of the rare mutation profiles, S531W, and a triple codon deletion (510–512) in northern Thailand. According to high accuracy, the RIF-RDp assay may render an easy-to-use, low-cost, and promising diagnostics of RR-TB in the future.

## 1. Introduction

Tuberculosis (TB) is a serious threat to global health that remains the second deadliest infectious disease after COVID-19. According to the WHO report, in 2019, an estimation of TB and death cases was 10.0 million and 1.4 million, respectively [1]. The emergence of drug-resistant TB (DR-TB) is a significant problem for TB control programs worldwide. In 2019, there were 465,000 incident cases of MDR-TB, and a global proportion of 78% of rifampicin (RIF) resistance TB had multidrug-resistant TB (MDR-TB) [1]. MDR-TB is caused by *Mycobacterium tuberculosis* (Mtb) strains that are simultaneously resistant to RIF and isoniazid (INH). It is also one of the most urgent and difficult challenges facing the global epidemic [2]. As RIF resistance is frequently concomitant to INH resistance, it was proposed as a surrogate marker for MDR-TB. Moreover, in 2018, the WHO introduced a short-course, all-oral regimen for the treatment of RIF-resistant (RR-TB) [3]. Thus, early detection of RIF resistance is vital to improve the management of MDR/RR-TB and eradicate TB from the world. However, a simple and accurate method for the diagnosis of RR-TB is needed for the successful treatment and prevention of DR-TB transmission.

Rifampicin (RIF), also known as rifampin, was introduced for use as an anti-TB drug in 1972 [4]. It serves as one of the major bactericidal antibiotics used to treat several types of bacterial infections, including TB. Nearly 95% of RIF-resistant mutations are associated with the *rpoB* mutation hot spot region so-called “the rifampicin resistance determining region (RRDR)” [5,6]. This 81-bp region ranges from codons 507 to 533 (27 amino acids) of the *rpoB* gene [7]. Rifampicin resistance found in Mtb is usually associated with a single-base substitution mutation within the *rpoB* gene that causes the single-nucleotide polymorphisms (SNPs). Four classes of SNPs are classified based on nucleotide base exchanges [8,9]. Class-I SNP involves transition substitutions (A/G→G/A or T/C→C/T). Transversion mutations of class-II, -III, and -IV SNPs contain A/C→C/A or T/G→G/T, C/G→G/C, and A/T→T/A changes, respectively.

Delay diagnosis of DR-TB impedes TB treatment and control. Conventional TB culture and drug susceptibility testing (DST) may take a long time (approximately 8−14 weeks) to obtain the Mtb drug susceptibility profiles. Although DST remains the standard method for the detection of DR-TB, its complexity, biosafety concern, the lack of facilities, and technical expertise are major limitations in low and middle-income countries (LIMCs). Several genotypic methods have been developed to offer an advantage over time-consuming phenotypic tests. However, the WHO recommended the Xpert MTB/RIF and line probe assays (LPAs) for diagnosis of DR-TB. The target coverage limitation, false positive results, and high rate of uninterpretable results are remaining problems for these methods [10,11]. Next-generation DNA sequencing is a high-throughput technique for the prediction of drug susceptibility among Mtb strains by detecting known and unknown mutations of antimicrobial resistance genes. However, the application of sequencing is prohibited by the need for robust software, massive output, and complex data analysis [12,13].

Real-time PCR with high-resolution melting curve analysis (HRM) has been applied as molecular detection of DR-TB [14,15,16]. HRM analysis provides the accurate identification of single-point mutations based on the different melting curves of PCR amplicons. However, only HRM analysis cannot detect all possible SNPs, especially class-III and -IV transversion mutations [17,18,19]. There are no changes in the number of hydrogen bonds of these two SNPs classes, thus, very little or no difference in melting temperature (Tm) between wild type and mutants. Recently, Anukool et al. [20] showed that the assays based on HRM, “RIF-RD” and “INH-RD”, have an overall good performance for the rapid detection of RR-Mtb and INH-R Mtb. However, the RIF-RD assay cannot detect H526D and D516V mutations among Mtb isolates from northern Thailand, which accounted for 10% and 3.3%, respectively [20]. Locked nucleic acids (LNAs) probes have previously been used in either real-time PCR or real-time PCR combined with HRM analysis (real time/HRM) to identify DR-TB [21,22]. Ramirez et al., 2010, reported the use of newly designed LNA probes in real time/HRM for the detection of the most common transversion mutations in MDR-TB [20,23]. LNA probes consist of modified oligonucleotide bases, which can increase the thermal stability, thus rendering high sensitivity detection of SNPs or single-point mutation and allowing a preferable signal-to-noise ratio. Therefore, this study designed two new LNA probes for the detection of the two most common class-III and -IV SNPs mutation found in Thailand [20,23]. Combining these probes with the previously developed assay, the new RIF-RDp assay was then established to improve the sensitivity of RR-TB detection. The potential of RIF-RDp assay efficiency was evaluated using blinded clinical isolates against standard phenotypic and genotypic assays, including a commercial real-time PCR test kit.

## 2. Materials and Methods

### 2.1. Clinical Mycobacterium Tuberculosis Strains and DNA Samples

For the optimization and validation of the RIF-RDp assay, reference strains of *Mycobacterium tuberculosis* ATCC 27294 (Mtb H37Rv), 20 known RIF-susceptible (RS), and 14 RIF-resistant (RR) strains were used (Table 1). One hundred and ten clinical Mtb isolates were included for the assay evaluation. DNA samples of all Mtb isolates were obtained from the TB regional laboratory, the Office of Disease Prevention and Control 1 (ODPC 1) Chiang Mai, Department of Disease Control, the Ministry of Public Health, Thailand. They were isolated from pulmonary TB patients at ODPC 1, which covers 15 hospitals in 8 provinces in northern Thailand (Chiang Mai, Chiang Rai, Phayao, Nan, Phrae, Lampang, Lamphun, and Mae Hong Son) and Chiangrai Prachanukroa Hospital (CPH) during 2015 to 2017. The identification of *M. tuberculosis* using the standard mycobacterial culture method. The Mtb isolates were recovered in 2% Ogawa medium at 37 °C for 4–8 weeks. Then, the genomic DNA was extracted using the extraction reagents of Anyplex^TM^ MTB/NTM Real-time Detection (Seegene, Republic of Korea) according to the manufacturer’s instructions. The DNA was stored at −20 °C before being used in the experiments.

### 2.2. The Design of H526D Probe and D516V Probe

Two new LNA probes were designed to detect specific SNP transversions, the H526D mutation (C→G, class-III SNP) and the D516V mutation (A→T, class-IV SNP) by modifying the target nucleotide specifically. Three LNAs were placed into the probe by using Integrated DNA Technologies (OligoAnalyzer™ Tool): one LNA at the specific target site as the center, and the remaining two are at every third base on each side. The probes were designed based on the introduction of SNPs in H37Rv Mtb (GenBank accession no. JX303332.1). The 16-bp H526D probe covers nucleotide positions 1346 to 1361 and is labeled with HEX at the 5’ end and a black hole quencher, BHQ1, at the 3’ end. The 18-bp D516V probe ranges from nucleotide position of 1316 to 1333 and is labeled with Cy5 at the 5’ end and BHQ2 at the 3’ end. These two probes were synthesized by Integrated DNA Technologies, Coralville, IA, USA.

### 2.3. The RIF-RDp Assay

The RIF-RDp assay was designed to identify RR-TB by detection of H526D and D516V mutations using LNA probes and other mutations on the *rpoB* gene using HRM analysis. The optimal conditions of the RIF-RDp assay, including annealing temperature, primer and probe concentrations were initially determined. Genomic DNA of Mtb H37Rv (wild type reference) and MD62 (RR-Mtb reference), and DNase free water (negative control) were used as control of the RIF-RDp assay. The 200-bp H526D and D516V synthetic DNA templates (Integrated DNA Technologies, Coralville, IA, USA) were also included as mutant controls in each run of the RIF-RDp. The assay was performed in a total of 25 µL per reaction: 0.8X LightCycler^®^ 480 HRM master mix including ResoLight high-resolution melting dye (Roche Diagnostics, Mannheim, Germany), 3.5 mM MgCl_2_, 0.3 µM of *rpoB* primers, 0.75 µM of each probe, and 100 ng of DNA template. The cycling profile for the assay was performed on the LightCycler^®^ 96 real-time PCR system (Roche Diagnostics, Basel, Switzerland) under the following conditions: 95 °C for 3 min, and 40 cycles of 95 °C for 10 s, 62 °C for 15 s, and 72 °C for 30 s. After amplification, the PCR products were analyzed by HRM analysis based on the following conditions: amplicons were heated at 95 °C and cooled down at 40 °C for 1 min each, and the temperature rose from 65 to 97 °C at a rate of 0.04 °C/s. 

### 2.4. Limit of Detection (LOD) of the RIF-RDp Assay

The LOD of the RIF-RDp assay (25-µL reaction) was performed in three separate reactions in duplication. To determine the assay LOD for the detection of the *rpoB* gene, the genome copies (gc) of Mtb H37Rv were calculated based on the amount of genomic DNA and Mtb H37Rv genome size [24]. Then, the Mtb H37Rv genomic DNA was 10-fold serially diluted from 2 × 10^6^ gc (10 ng) to 2 × 10^1^ gc (100 fg). To obtain 1 × 10^2^ gc (500 fg), 2 × 10^2^ Mtb H37Rv gc was two-fold diluted. Then, 2 × 10^6^, 2 × 10^5^, 2 × 10^4^, 2 × 10^3^, 2 × 10^2^, 1 × 10^2^, 2 × 10^1^ H37Rv gc were used as DNA template for RIF-RDp LOD testing. To determine the LOD for the detection of H526D and D516V mutations in HEX and Cy5 channels, the gc of H526D and D516V synthetic DNA were calculated and the dilutions of the DNA were prepared in the same manner from 2 × 10^6^ to 2 × 10^1^ gc. 

### 2.5. Evaluation of the RIF-RDp Assay

The RIF-RDp assay was performed on a total of 110 blinded Mtb isolates. These isolates were also analyzed by phenotypic DST, DNA sequencing, and a commercial kit (Anyplex™ II MTB/MDR assay, Seegene, the Republic of Korea).

#### 2.5.1. Drug Susceptibility Testing (DST)

For the standard DST, Mtb isolates were performed by the proportion method according to the Clinical and Laboratory Standards Institute (CLSI) document M24-A2 [25] using Middlebrook 7H10 agar and incubated at 37 °C, ambient air. Isolates were considered resistant when ≥1 percent of colony growth was observed on the agar medium containing the critical concentrations of RIF at 1.0 μg/mL. The Mtb H37Rv was used as a quality control strain.

#### 2.5.2. DNA Sequencing

The 543-bp *rpoB* gene amplicons were amplified by PCR primers previously described by Ramirez et al., 2010 [22], covering the RRDR. Before sequencing, amplicons were purified with NucleoSpin^®^ Gel and PCR Clean-up (MACHEREY-NAGEL GmbH & Co. KG, Düren, Germany). DNA sequencing was performed by Bioneer Co., Ltd., Daejeon, South Korea using the ABI 3730*xl* DNA analyzer (Thermo Fisher Scientific, CA, USA). The mutation profiles were examined by Bioedit version 7.2 [26].

#### 2.5.3. Multiplex Real-Time PCR Commercial Kit

The Anyplex^TM^ II MTB/MDR Detection assay (commercial kit) was performed using the CFX96 real-time PCR System (Bio-Rad, Hercules, CA, USA). The 20-µL reaction mixture in a multiplex real-time PCR commercial kit, including 15 µL of PCR master mix and 5 µL of DNA sample, was analyzed according to the manufacturer’s protocol. Results were automatically interpreted using Seegene Viewer software (Seegene Inc., Seoul, Republic of South Korea) following the presets outlined by the manufacturer. 

### 2.6. Statistical Analysis

For HRM analysis, the melting temperature (Tm) of the *rpoB* amplicons was analyzed. The Tm difference (∆Tm) of each sample was then calculated by subtraction of its Tm from the Tm of Mtb H37Rv amplicons. Based on the analysis of ∆Tm of all samples, the Survival Analysis in XLSTAT (Addinsoft, New York, NY, USA) was used to generate the receiver operating characteristic (ROC) curve for establishing the ∆Tm cutoff value for accurate differentiation between RR-Mtb and RS-Mtb strains [27]. The sensitivity, specificity, positive predictive value (PPV), and negative predictive value (NPV) were determined to evaluate the performance of the RIF-RDp assay by comparing the efficiency with phenotypic DST, DNA sequencing, and Anyplex™ II MTB/MDR Detection assay. Moreover, the Cohen’s kappa agreement test between the assays was calculated using SPSS software v. 22.0.

## 3. Results

### 3.1. Optimization and the Validation of the RIF-RDp Assay

**Figure 1:**
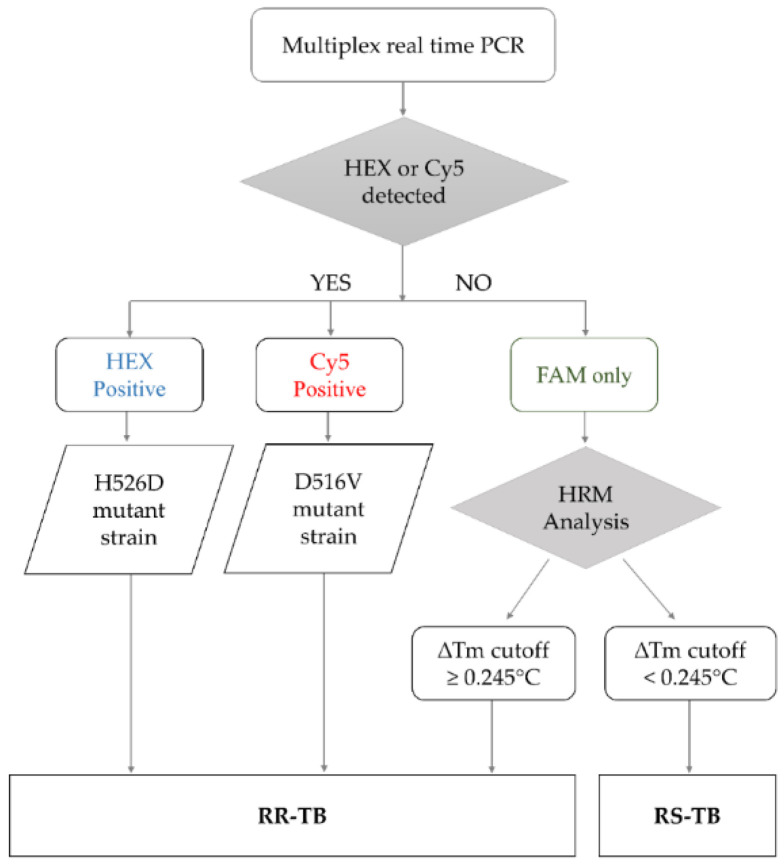
The algorithm for result interpretation of the RIF-RDp assay. H526D and D516V mutations can be detected in the HEX channel and Cy5 channel, respectively, and identified as RR-TB. Other mutations were differentiated based on the ∆Tm cutoff value. If the ∆Tm cutoff value is ≥0.245 °C, it was interpreted as rifampicin-resistant tuberculosis (RR-TB) and if it is <0.245 °C, it was identified as rifampicin-susceptible tuberculosis (RS-TB).

The RIF-RDp assay was set up based on the HRM analysis combined with LNA probes to identify RR-Mtb strains. The results interpretation algorithm was shown in Figure 1. If either H526D or D516V mutation was detected in the HEX or Cy5 channels (positive result), the result can be interpreted as resistant (R). When none of H526D or D516V was detected, other *rpoB* mutations were determined based on the ∆Tm cutoff value to differentiate RR-Mtb from RS-Mtb strains.

**Figure 2:**
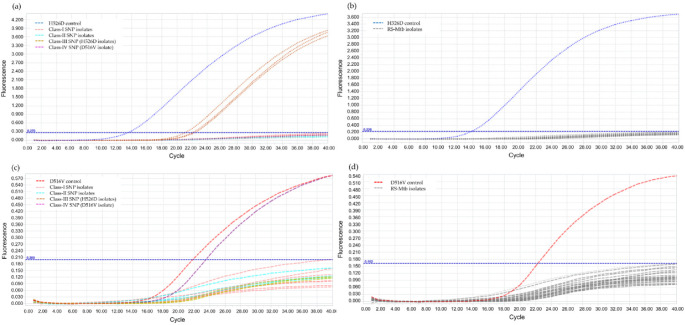
Validation of the RIF-RDp assay for detection of H526D and D516V mutant Mtb strains using 14 rifampicin-resistant *M. tuberculosis* (RR-Mtb) strains (7, 3, 3, and 1 strain of Class-I, Class-II, Class-III (H526D), and Class-IV SNP (D516V)), 20 rifampicin-susceptible *M. tuberculosis* (RS-Mtb) strains, H526D and D516V mutant controls, and *M. tuberculosis* H37Rv reference strain. The fluorescence signal detected in the HEX channel (**a**,**b**), or Cy5 channel (**c**,**d**) illustrates the detection of H526D or D516V mutant Mtb strains, respectively.

The annealing temperature was determined using PCR gradient, by increasing 2 °C within a range of 56–66 °C and the result showed the optimal annealing temperature at 62 °C. For the validation of LNA probes, RIF-RDp correctly distinguished H526D and D516V mutant strains from all RS-Mtb strains and RR-Mtb strains containing mutation profiles other than H526D and D516V (Figure 2).

### 3.2. LOD of the RIF-RDp Assay

**Figure 3:**
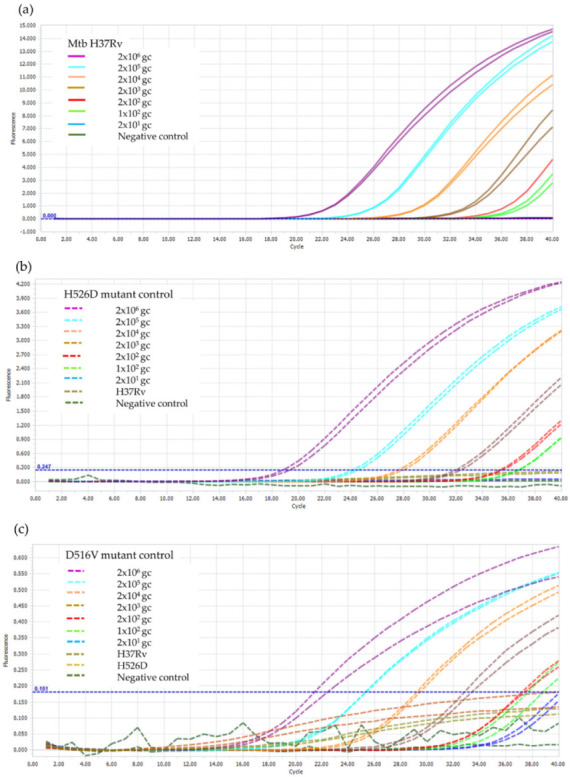
LOD determination of the RIF-RDp assay for detection of (**a**) *rpoB* gene, (**b**) H526D mutation, and (**c**) D516V mutation. The duplicate DNA samples of *M. tuberculosis* H37Rv (H37Rv), and the H526D and D516V mutant controls using 2 × 10^6^ to 2 × 10^1^ genome copies (gc) of Mtb H37Rv and negative control (sterile distilled water).

It was found that the LOD of the RIF-RDp assay for the detection of *rpoB* gene, H526D, and D516V mutations was 100 genome copies of Mtb H37Rv, which is equivalent to 100 bacilli of Mtb as shown in Figure 3.

### 3.3. Evaluation of the RIF-RDp Assay and ROC Curve Analysis

**Figure 4:**
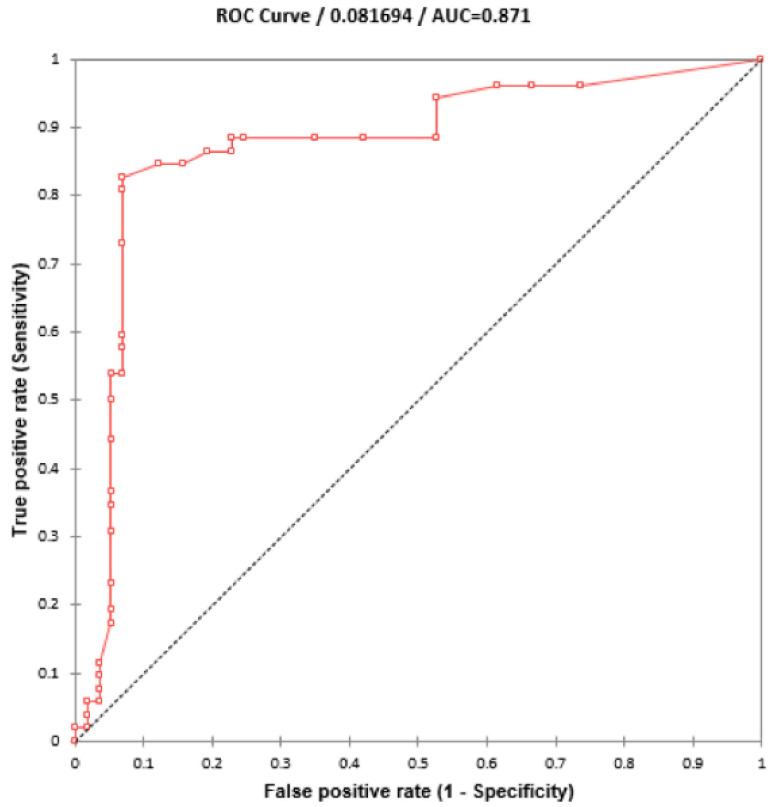
ROC curve for diagnosis of RR-TB based on HRM analysis. The ∆Tm cutoff value at 0.245°C was established to differentiate between rifampicin-resistant *M. tuberculosis* (RR-Mtb) and rifampicin-susceptible *M. tuberculosis* (RS-Mtb).

**Figure 5:**
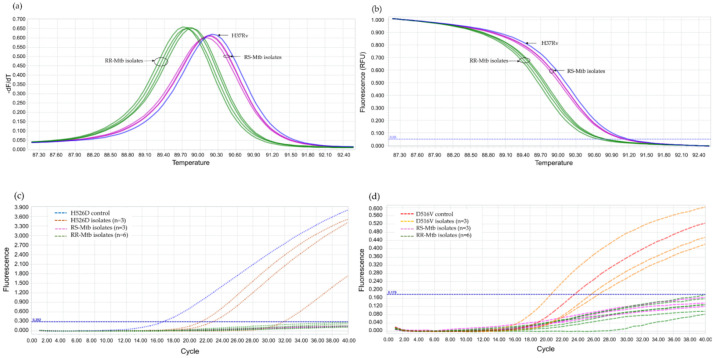
The performance of the RIF-RDp assay in the detection of rifampicin-resistant *M. tuberculosis* (RR-Mtb). The normalized melting peaks (**a**), and normalized melting curves (**b**) clearly distinguished RR-Mtb from rifampicin-susceptible *M. tuberculosis* (RS-Mtb) isolates based on HRM analysis. RR-Mtb strains harboring H526D or D516V mutation were detected in the HEX (**c**) or Cy5 channels (**d**), respectively.

Using 110 blinded clinical Mtb isolates, the RIF-RDp assay was evaluated versus DST (55 RR- and 55 RS-Mtb isolates), DNA sequencing, and Anyplex^TM^ II MTB/MDR Detection assay. Using the ∆Tm of all isolates, the receiver operating characteristic (ROC) curve established the ∆Tm cutoff value at 0.245 °C to differentiate between RR-TB and RS-TB. The ROC curve showed the area under the curve (AUC) at 0.9, which indicated that this assay was highly accurate for the detection of *rpoB* mutations (Figure 4). Based on this cutoff value, the RIF-RDp assay efficiently distinguished RR-Mtb from RS-Mtb isolates (Figure 5a,b). H526D and D516V mutations were detected in the HEX and Cy5 channels (Figure 5c,d). The sensitivity, specificity, PPV, and NPV of the RIF-RDp assay and commercial kit against DST and DNA sequencing were shown in Table 2. The RIF-RDp assay showed high sensitivity and accuracy, that was comparable to the commercial kit and DNA sequencing. The RIF-RDp assay showed sensitivity, specificity, PPV, and NPV at 96.36, 100, 100, and 96.49% while the commercial kit achieved 94.55, 100, 100, and 94.83% when compared to DNA sequencing. In addition, the RIF-RDp assay had almost perfect agreement by Cohen’s Kappa coefficients with the commercial kit (κ = 0.95, 95%CI, 0.89–1), DNA sequencing (κ = 0.96, 95%CI, 0.92–1), and DST (κ = 0.93, 95%CI, 0.86–1).

In addition, to evaluate its potential use in Thailand, the price per test of the new assay was determined and compared to the commercial molecular tests available in the country. The cost of the RIF-RDp assay was assessed based on the expense on DNA extraction and HRM real time PCR. The machine maintenance, electricity, and personnel expenses were not included. The DNA extraction of Mtb isolates using AnyplexTM MTB/NTM Real-time Detection (Seegene, Republic of Korea) cost 200 Thai bahts (THB) per sample. The cost of a real-time PCR reaction (the HRM reaction mixture, one pair of primers, and 2 LNA probes) was 65 Thai bahts (THB) per 25-µL reaction. The overall cost was 265 THB, which were approximately 7.18 US Dollars ($) (the foreign exchange rates at 36.968 THB/US Dollar, as of 20 September 2022, https://www.bot.or.th). Anyplex™ II MTB/MDR assay and DNA extraction cost 42,500 THB per 50 samples. Therefore, it cost 850 THB ($23.01) per test. The price of Gene Xpert MTB/RIF cartridges was 500 THB each ($13.54), and LPA test including DNA extraction by the same kit (200 THB)/sample) and the GenoType MTBDRplus (Hain Lifescience, Nehren, Germany) (1800 THB) cost 2000 THB ($54.16). 

## 4. Discussion

In this study, we developed a new assay RIF-RDp that incorporated the fluorophores-labeled LNA probes with the real-time PCR and HRM analysis to detect RR-TB that is associated with *rpoB* mutations in clinical Mtb isolates. This new assay successfully detected the class-III and –IV SNPs; H526D (C→G), and D516V (A→T) commonly found in northern Thailand [28]. Due to a slight shift of Tm detected in these SNPs, they were undetectable by the HRM-based assays previously reported [17,18,19,20]. This newly developed RIF-RDp assay showed a higher sensitivity compared to the previous RIF-RD assay [20]. The RR- and RS-Mtb isolates can be differentiated based on the accurate ∆Tm cutoff value. The LOD of the RIF-RDp assay was equivalent to 100 genome copies of Mtb H37Rv, which is lower than that of the RIF-RD assay (210 genome copies) [20] but higher than that of the Anyplex™ II MTB/MDR (20 genome copies).

The performance of the developed RIF-RDp assay on 110 blinded clinical isolates showed 94.55% sensitivity and 98.18% specificity when compared to DST. Among the blinded samples, we found that four samples showed discordant results. Three RR-Mtb isolates were identified as susceptible (negative) by RIF-RDp. The DNA sequencing result showed that two of three isolates contained *rpoB* mutation, one possessed class-II SNP (H526P, A→C) and another class-III SNP (S531W, C→G) mutations. Both S531W and H526P mutant samples generated the ∆Tm below the cutoff value though H526P mutation is class-II SNP. This may be caused by a low level of amplification due to inadequate DNA quality. This isolate was also identified as RIF susceptible by the commercial kit because H526P is not included in the assay targets. The last isolate showed no mutation by DNA sequencing of the 543-bp (codons 422 to 603) of *rpoB* and was also identified as susceptible by the commercial kit. This conferred RIF resistance may be resulting from other mutations outside the *rpoB*-target region or alternative mechanisms such as efflux-related mechanisms [29,30] or drug modifying enzymes [31]. In one isolate with a false positive result, DNA sequencing confirmed that it contained nonsense mutation with class-I SNP (CCC→CCT) at codon 535.

In this study, the most predominant mutations at codons 531, 526, and 516 accounted up to 88.8% of all RR-Mtb strains. Fifteen mutation profiles were detected by DNA sequencing. Interestingly, an Mtb isolate with a rare mutation profile, S531W, was firstly found in northern Thailand. S531W is a TCG→TGG substitution mutation that was associated with an MDR-TB outbreak in London, England during 1995–2004. This mutant isolate was firstly found in an MDR-TB developed patient with poor compliance therapy and was then detected in a primary MDR-TB patient [32]. Nevertheless, the S531W mutation was also the second most prevalent in Santa Catarina state, southern Brazil at 20.8% (11/53) [33].

To the best of our knowledge, this is the first report for triple codon deletion at codons 510, 511, and 512 in Mtb confirmed by DNA sequencing. Since the deletion causes the loss of three amino acids (glutamine, leucine, and serine), thus, conferring RIF resistance that correlates with DST. In 2008, Prammananan T, et al. reported that two MDR-Mtb isolates in Thailand harbored double codon deletion: one at codons 513 and 514; and another at codons 518 and 519 [23]. A study reported the detection of two RR-Mtb isolates in India that contained deletion at codon 518, could not be detected by LPAs due to the overlapping between the WT4 and WT5 probes [34]. In Brazil, one MDR-Mtb isolate containing four codon deletions at 516-518 has been reported in 2000. This study found that some codon deletions were combined with other single-point mutations, for example, one MDR-Mtb isolate had triple codon deletion from codons 514 to 516 and a point mutation at codon 513, and one isolate had triple codon deletion at codon 524, 525, and 526 and one codon deletion at codon 527 [35].

The RIF-RDp assay and the commercial kit showed equal specificity (100%) when compared to DNA sequencing. Although the RIF-RDp assay and the commercial kit are based on real-time PCR and can be performed up to 96 reactions in a single run, there are advantages and disadvantages to each one. The RIF-RDp assay rendered less time for detection. It took approximately 2.5 h from the preparation of reaction mixture to the analysis and interpretation of the result while the Anyplex™ II MTB/MDR assay took up to 3.5 h. If it is performed routinely, the RIF-RDp may need a 1-day total turnaround time (TAT) to report the results, which is comparable to other available Nucleic Acid Amplification tests (NAATs). However, the result interpretation of Anyplex^TM^ II MTB/MDR is more convenient due to an automatic data analysis. The RIF-RDp assay requires the calculation of ΔTm between test samples and wild type reference to distinguish between RR-TB and RS-TB. A faster and more automated analysis and interpretation by integrating the user-friendly software for RIF-RDp like a viewing program used for Anyplex™ II MTB/MDR assay shall be innovated in future. In addition, the cost of the RIF-RDp assay per test using a 25-µL reaction is only US$7.18, which is about 2–3 times cheaper compared to the commonly used molecular tests in Thailand, Anyplex™ II MTB/MDR assay ($23.01/850 THB), Gene Xpert MTB/RIF ($13.54/500 THB), and LPAs ($54.16/2000 THB). Due to the high-cost equipment and cartridge, the use of Xpert MTB/RIF is still limited. LPA provides profiles of *rpoB* mutations but it requires a complicated process with PCR and DNA hybridization. Therefore, it takes a longer TAT, approximately 2 days. As there is a need for a real-time PCR machine and well-trained staff in RIF-RDp assay, it may be used as a candidate RR-TB assay in the reference or regional laboratories. 

Since the RIF-RDp was developed based on HRM analysis that requires a high quality of DNA samples. The low quality of DNA samples can cause a low amplification of target that affects the melting process and may cause false negative result. This new assay was only tested in *M. tuberculosis* isolates cultured from clinical specimens. Further study of the direct detection of RR-Mtb in patient specimens like sputum is still needed to speed up the diagnosis of RR-TB. Only 3 H526D and 1 D516V mutant isolates and 14 isolates with Class-I-IV SNP mutation were available for the validation of the designed LNA probes and the evaluation of RIF-RDp. Testing with more mutant isolates harboring H526D and D516V mutations, and also other mutations, may be necessary to assure the reliability of this assay. Even though, there are several rare transversion mutation class-III and -IV SNPs have been reported [28]; herein, the RIF-RDp assay was designed to detect the two most common class-III and IV mutations found in northern Thailand. Since all RIF resistance-related mutations that occur outside the RRDR are not covered by the RIF-RDp assay, thus it cannot replace the standard DST method. However, the RIF-RDp assay can detect the majority of known, unknown, and rare mutations within RRDR that are associated with *rpoB* mutation in RR-TB, making it superior to other available molecular assays in terms of assay spectrum.

## 5. Conclusions

We developed the RIF-RDp assay by combining LNA probes with HRM analysis to detect RR-TB with high sensitivity and specificity. The newly designed probes showed 100% specificity for detection of the H526D and D516V mutant strains, which were not identified by HRM analysis alone. This assay is easy to perform, cost-effective, and beneficial for the diagnosis of RR-TB. The application of this assay at the reference or the regional laboratories in Thailand is proposed to improve the capacity in preventing treatment failure and control the future spread of drug-resistant TB.

## Figures and Tables

**Table 1:** *Mycobacterium tuberculosis* isolates used in the validation process of the RIF-RDp assay.

Sample Code	Mutation Profile	SNP Type	Source
*Rifampicin-resistant M. tuberculosis (n = 14)*			
CM1	S531L (TCG→TTG)	Class-I (*n* = 7)	ODPC 1 *
CM2	S531L (TCG→TTG)		ODPC 1
CM3	S531L (TCG→TTG)		ODPC 1
CM4	S531L (TCG→TTG)		ODPC 1
CM5	H526Y (CAC→TAC)		ODPC 1
CR1	H526C (CAC→TGC)		CPH **
CM6	S522L (TCG→TTG)		ODPC 1
CM7	L511P (CTG→CGG)	Class-II (*n* = 3)	ODPC 1
CR2	Q513P (CAA→CCA)		CPH
CR3	H526P (CAC→CCC)		CPH
CM8	H526D (CAC→GAC)	Class-III (*n* = 3)	ODPC 1
CR4	H526D (CAC→GAC)		CPH
CR5	H526D (CAC→GAC)		CPH
CR6	D516V (GAC→GTC)	Class-IV (*n* = 1)	CPH
*Rifampicin-susceptible M. tuberculosis* *(n = 20)*			
CM9-CM28	No mutation		ODPC 1

* ODPC 1: Office of Disease Prevention and Control ** CPH: Chiangrai Prachanukroa Hospital.

**Table 2:** Comparison of the performance between the RIF-RDp assay and Anyplex™ II MTB/MDR in the detection of rifampicin-resistant tuberculosis against drug susceptibility testing (DST) and DNA sequencing method.

Assays		RIF-RDp		PPV, NPV * (95% CI)	Anyplex^TM^ II MTB/MDR		PPV, NPV * (95% CI)
		R	S		R	S	
**DST**	**R**	52	3	98.11% (88.16–99.73)	52	3	100%
	**S**	1	54	94.74% (85.69–98.19)	0	55	94.83% (85.92–98.22)
**Sensitivity, Specificity** **(95% CI)**		94.55% (84.88–98.86)	98.18% (90.28–99.95)		94.55% (84.88–98.86)	100% (93.51–100)	
**DNA** **sequencing**	**R**	53	2	100%	52	3	100%
	**S**	0	55	96.49% (87.58–99.08)	0	55	94.83% (85.92–98.22)
**Sensitivity, Specificity** **(95% CI)**		96.36% (87.47–99.56)	100% (93.51–100)		94.55% (84.88–98.86)	100% (93.51–100)	

* PPV: positive predictive value, NPV: negative predictive value, 95% CI: confidence interval.

## Data Availability

Not applicable.
